# Recommendation Based on Trust Diffusion Model

**DOI:** 10.1155/2014/159594

**Published:** 2014-06-09

**Authors:** Jinfeng Yuan, Li Li

**Affiliations:** Faculty of Computer and Information Science, Southwest University, Chongqing 400715, China

## Abstract

Recommender system is emerging as a powerful and popular tool for online information relevant to a given user. The traditional recommendation system suffers from the cold start problem and the data sparsity problem. Many methods have been proposed to solve these problems, but few can achieve satisfactory efficiency. In this paper, we present a method which combines the trust diffusion (DiffTrust) algorithm and the probabilistic matrix factorization (PMF). DiffTrust is first used to study the possible diffusions of trust between various users. It is able to make use of the implicit relationship of the trust network, thus alleviating the data sparsity problem. The probabilistic matrix factorization (PMF) is then employed to combine the users' tastes with their trusted friends' interests. We evaluate the algorithm on Flixster, Moviedata, and Epinions datasets, respectively. The experimental results show that the recommendation based on our proposed DiffTrust + PMF model achieves high performance in terms of the root mean square error (RMSE), Recall, and *F* Measure.

## 1. Introduction

With the rapidly growing amount of information available on the WWW, recommender systems become a popular way to help users select relevant information on the Internet. Personalized recommendation adopts knowledge discovery techniques such as data mining and machine learning to discover user interests according to user behavior and then to make recommendations [1, 2]. Typically, collaborative filtering (CF) is the most successful and widely used recommendation technique [3, 4]. CF makes recommendation according to the assumption that users who have the similar performances would like to choose the similar items. Despite its popularity and success, the performance of CF is significantly limited by the “data sparsity” and “cold start” [3, 5].

In view of these limitations, many scholars have recently integrated trust relationship among users into the recommendation system [6–10]. Trust-based recommender systems utilize a social network augmented with trust ratings, known as a trust network, to generate recommendations for users based on people they trust. These systems can deal with the trust relations between users, which cannot be well handled in traditional CF-based recommendations, to support the recommendation process. However, these trust-based methods work to some extent and further have several other inherent weaknesses. Firstly, the sparsity of trust network. In recommender systems, the number of users is often very large, but in the process of real world recommendations, the number of direct transactions between users is small. So the number of direct trust relationships established by the limited number of transactions is also very little, which leads to the fact that the direct trust relationships can only play a small role in the process of the recommendation. Many trust-based recommendation algorithms only obtain trust values from preexisting social links between users [6–8] and do not consider indirect trust relationships. Secondly, the dynamics of the trust network. In the trust network, the trust relationships between users are not static, but dynamically change over time or other events. The change of the trust relationships may result in change of recommendation results. Therefore, it is more feasible to use dynamic trust network [8, 10, 11]. Thirdly, how to integrate the trust model with the recommendation system. Most recommendation algorithms [12–14] are based on the traditional probabilistic matrix factorization model and fusion between user-item matrix and social relationships by sharing a potential low dimensional user characteristic matrix. These methods can only learn few effective characteristics. This disadvantage not only causes lack of interpretability in the model, but also affects the quality of the recommendation.

Aiming at effectively overcoming the above limitations and modeling recommender systems more accurately this paper presents a new recommendation method based on trust diffusion mechanism (DiffTrust + PMF). The contributions of this paper are as follows. (1) According to the diffusion theory in economics, we improve the trust diffusion model (DiffTrust) suitable for collaborative filtering recommendation systems. DiffTrust makes full use of the direct trust relationship between users, to derive the indirect trust relationship between users through a certain trust propagation rules. (2) We employ the probabilistic matrix factor (PMF) model to put DiffTrust into the process of the recommendation. DiffTrust + PMF has the following characteristics. On one hand, this method matches more trust users for the current user through DiffTrust model and fully digs up more new trust relationships between users and is used for recommendation service, which perfectly solves the data sparsity of the trust network. On the other hand, DiffTrust + PMF combines the users' tastes with their trusted friends' interests through a set of parameters. This consideration can truly reflect the recommendation process in real life. (3) Through experiments on three data sets— Flixster, Moviedata, and Epinions datasets—we found that the proposed DiffTrust + PMF method can improve performance much more than other existing algorithms. In other words, the combination of DiffTrust and probabilistic factor model for recommendation outperforms all others in both the trust-based recommenders and based-PMF (probabilistic matrix factorization) recommenders.

The remainder of the paper is organized as follows.  Section 2 discusses our method using DiffTrust + PMF.  Section 3 evaluates the procedures experimentally followed by further discussion of relevant issues associated with models used in the paper. The related work is reviewed in Section 4. Finally, Section 5 concludes the paper.

## 2. New Method for Recommendation

In this section, we first describe recommendation problem based on trust network in Section 2.1 and then provide the solution in Sections 2.2 and 2.3.

### 2.1. Problem Description

**Figure 1:**
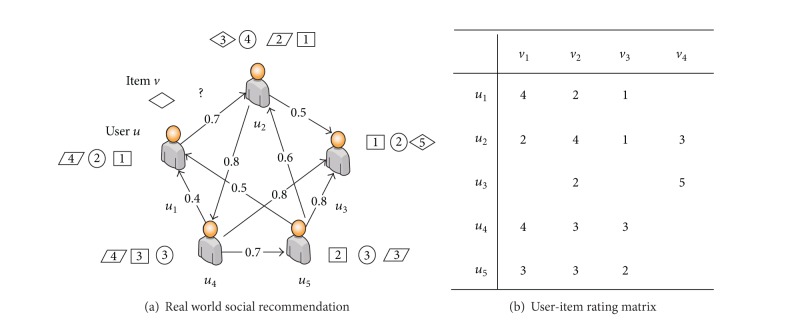
Example for trust-based recommendation problems.

The problem we study in this paper is different from traditional recommender systems, because the latter normally only utilize the information of the user-item rating matrix. In this paper, we will also incorporate the social trust relations among users to improve recommender systems.  Figure 1(a) shows the social process of recommendation in the real world. The users are connected with edges, and each edge is associated with a weight in the range [0,1] to indicate how much user *u*_*i*_ knows or trusts user *u*_*k*_; zero means no trust and one means full trust. Each user also rated some items on a 5-point integer scale to express the extent of the favor of each item. The source user *u* wants a prediction on the target item *v*. The recommendation scenario includes two central elements: the friends network and the favors of these friends, which can essentially be illustrated by the examples of social network graph in Figure 1(a) and user-item matrix in Figure 1(b), respectively.

In the real word, we consider an *m* × *n* rating matrix *R* describing *m* users' numerical ratings on *n* items, where *r*_*ij*_ represents the rating of user *i* for item *j*. In most online systems, *r*_*ij*_ is the K-point integer (normally, 1, 2, 3, 4, and 5 represent “very bad,” “bad,” “neutral,” “good,” and “very good,” resp.).

We define a directed trust-based graph *G* = (*U*, *E*) among users, where the vertex set *U* = {*u*_*i*_}_*i*=1_^*m*^ and edge set *E* correspond to all the users in a social trust network and the trust relations between users, respectively. For a pair of nodes, *u*_*i*_ and *u*_*k*_, the weight associated with an edge from *u*_*i*_ to *u*_*k*_ is denoted as *t*_*ik*_ ∈ [0,1], which represents how much a user *u*_*i*_ trusts. Let *T* = {*t*_*ik*_} denote the *m* × *m* trust matrix of *G*. Note that matrix *T* is an asymmetric matrix, since in a trust-based social network, user *u*_*i*_ trusting *u*_*k*_ does not necessary indicate user *u*_*k*_ trusts *u*_*i*_.

The goal we study in this paper is how to effectively and efficiently predict the missing values of the user-item matrix by employing the ratings expressed by trusted friends. The most existing trust-based methods [6–8] only use explicit trust information explicitly indicated by users to generate recommendations, while implicit trust relationship is ignored. We employ diffusion trust model to explore the processes which the trust has cultivated, which can infer the implicit relationship of the trust network. The detailed process will be introduced in the next section.

### 2.2. Novel Trust Diffusion Model

**Figure 2:**
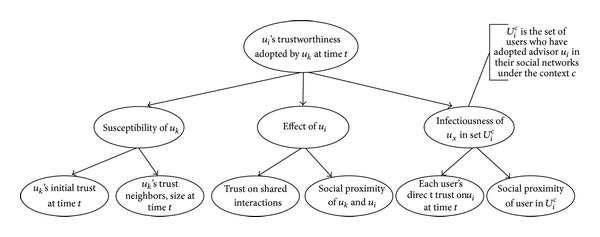
Trust Diffusion Model.

In order to present the concepts involved for predicting the trustworthiness, we improve the existing DiffTrust method [15] inspired by the individual-level framework of the diffusion theory [16]. According to the diffusion theory in social science, an advisor's trust building among users is considered as a diffusion process, involving the purposes of evaluating and using trust of the user (why), the ways and channels of inducing trust towards the user (how), and the induced trust degree (at what rate) varying over time for other users or other contexts. Our improved trust diffusion model emphasizes the dynamics and evolutionary characteristics of trust. That is, the trustworthiness of an advisor may be perceived differently by different users, which is dependent on the environment and embedded with a specific context.  Figure 2 shows the DiffTrust model.

As shown in Figure 2, the DiffTrust models the trustworthiness of users by incorporating three major parts:* intrinsic tendencies*, which refers to user *u*_*k*_' intrinsic nature without any direct or indirect experience with user *u*_*i*_, which includes user *u*_*k*_' static initial trust at time *t* (*T*_*k*_^0^ ∈ [0,1]), and the number of *u*_*k*_'s trust neighbors in the social network at a specific time.* Direct connections*, represents *u*_*i*_' direct influence on *u*_*k*_. Two factors are considered: direct trust based on shared interactions between *u*_*i*_ and *u*_*k*_ and social proximity of *u*_*i*_ with respect to *u*_*k*_, denoted by *S*_*i*_^*k*^.* Contagion influence*, the impact from the users who have adopted *u*_*i*_, consists of two factors: each user's direct trust on *u*_*i*_ at time *t* and social proximity of *u*_*i*_ and user in *U*_*i*_^*c*^. *U*_*i*_^*c*^ is the set of users who have adopted advisor *u*_*i*_ in their social networks under the context *c*. We employ the existing largest ontology—the LinkedData ontology [17]—to describe the context. The context *c* (*c* ∈ {*c*_1_, *c*_2_,…, *c*_*m*_}), where *m* is the number of contexts in the system) is represented by a set of interacted items. Our goal is to compute the *u*_*i*_'s trustworthiness adopted by *u*_*k*_ at time *t* and under each context *c* (*c* ∈ {*c*_1_, *c*_2_,…, *c*_*m*_}), as *T*(*u*_*k*_, *u*_*i*_, *t*, *c*).

It's worth noting that we consider trust to be multidimensional. The trust relationships between users take place under certain conditions, which is modeled as multidimensional features—intrinsic tendencies, direct influence, and contagion influence. Users have different levels of expertise in different domains; each user has different opinions about other users' expertise in different conditions. That is, one user may be trustworthy, completely ignorant, or untrustworthy about others. In this paper, we focus on trust and unknown relationships. The discussion of trust (friendship), distrust (enmity), and unknown within the diffusion model was introduced in [18]. Further discussion of trust, distrust, and unknown within the trust diffusion model will be exploited in the future.

The computational steps of trustworthiness are as follows.

**Step 1 (compute the effect of direct connections)**

Based on the previous shared interactions of user *u*_*k*_ and advisor *u*_*i*_, at time *t*_0_, *u*_*k*_'s direct trust towards *u*_*i*_ under context *c* is denoted as *DT*(*u*_*k*_, *u*_*i*_, *t*_0_, *c*). At time *t*, *u*_*k*_ and *u*_*i*_ have a new shared interaction with the same item, denoted as *v*_*u*_*k*_ under the context *c*_*u*_*k*_ and *v*_*u*_*i*_ under the context *c*_*u*_*i*_, respectively. Then, based on the new shared interaction at time *t* under context *c*, we update user *u*_*k*_'s direct trust towards user *u*_*i*_, as follows:

(1) $$\begin{array}{c} DT\left(u_{k},u_{i},t,c\right)=\frac{DT\left(u_{k},u_{i},t_{0},c\right)\lambda^{\left(t-t_{o}\right)}+DI\left(v_{u\_k},v_{u\_i},t\right)}{1+\lambda^{\left(t-t_{o}\right)}}, \end{array}$$

where 0 < *λ* ⩽ 1 is a time decay factor for user to decrease the effect of old shared interactions between *u*_*k*_ and *u*_*i*_. *DI*(*v*_*u*_*k*_, *v*_*u*_*i*_, *t*) is the similarity between *v*_*u*_*k*_ and *v*_*u*_*i*_; the formula is as follows:

(2) DI(vuk,vui,t)=(1−|vu_k−vu_i|)×S(cuk,cui)×S(cuk,c),

where *S*(*c*_*u*_*k*_, *c*_*u*_*i*_) is the similarity between contexts *c*_*u*_*k*_ and *c*_*u*_*i*_, and *S*(*c*_*u*_*k*_, *c*) is the similarity between contexts *c*_*u*_*k*_ and *c*. The context *c*_*u*_*k*_ is the set of the items interacted by user *u*_*k*_, and *c*_*u*_*i*_ is the set of items interacted by advisor *u*_*i*_. We assume context *c*_*u*_*k*_ = 〈*c*_*u*_*k*_^1^, *c*_*u*_*k*_^2^,…, *c*_*u*_*k*_^*n*^〉 and context *c*_*u*_*i*_ = 〈*c*_*u*_*i*_^1^, *c*_*u*_*i*_^2^,…, *c*_*u*_*i*_^*n*^〉, where *n* is the number of items interacted by user. The context similarity is computed according to paper [19].

**Step 2 (computing contagious influence)**

Based on the diffusion theory, we can employ the weighted average of trust evaluation on user *u*_*i*_ from each user *u*_*x*_ ∈ *U*_*i*_^*c*^ to model contagious influence of users in *U*_*i*_^*c*^, denoted as

(3) $$\begin{array}{c} CI\left(u_{k},U_{i}^{c},t\right)=\frac{\sum_{u_{x}\in U_{i}^{c}}DT\left(u_{k},u_{x},t,c\right)\times S_{x}^{k}}{\sum_{u_{x}\in U_{i}^{c}}S_{x}^{k}}, \end{array}$$

where *S*_*x*_^*k*^ is *u*_*x*_'s social proximity with respect to user *u*_*k*_, which is computed according to socially spatial information [20].

**Step 3 (combining the three factors)**

By considering the three factors: susceptibility, direct connections, and contagious influence, the *u*_*i*_'s trustworthiness is adopted by *u*_*k*_ at time *t* under context *c*, denoted as *T*(*u*_*k*_, *u*_*i*_, *t*, *c*). The previous method [15] computes the *T*(*u*_*k*_, *u*_*i*_, *t*, *c*) through the linear combination of three factors.

Consider

(4) T(uk,ui,t,c)=ω1Tk0+ω2DT(uk,ui,t,c)+ω3CI(uk,Uic,t),

where *ω*_1_, *ω*_2_, and *ω*_3_ are the weight of effect from intrinsic tendencies, effect from direct connections, and contagious influence, respectively. The specific calculation of weights is introduced later.

*Main Step for Improvement*. Our improved method allows exponential decline in the additive model according to the diffusion theory [16], which better accommodates the introduction of temporal heterogeneity.

Consider

(5) T(uk,ui,t,c) =exp⁡(ω1Tk0)  +∑s∈φ(t)exp⁡[ω2DT(uk,ui,t,c)+ω3CI(uk,Uic,t)+λ(t−ts)],

where *ω*_1_, *ω*_2_, and *ω*_3_ are same as in (4). *λ* is same with (1). *φ*(*t*) contains all spatially relevant individuals adopted before *t*. *t* ^*s*^ is the adoption time of *s*th member of *φ*(*t*).

*ω*_2_ can be measured as follows:

(6) $$\begin{array}{c} \omega_{2}=\{\begin{array}{cc} \frac{N_{u_{k}}^{t}}{N_{\min}}, & \text{if}\;N_{u_{k}}^{t}\leq \frac{N_{u_{k}}^{t}}{N_{\min}},\\ 1, & \text{otherwise}. \end{array} \end{array}$$

*N*_*u*_*k*__^*t*^ is the number of users who are trusted by user *u*_*k*_ in time *t* and *N*_min⁡_ is the minimum number of neighbors needed for user *u*_*k*_ to be confident about their own evaluation, which can be determined by an acceptable level of error and a confidence measurement in [21] as follows:

(7) $$\begin{array}{c} N_{\min}=-\frac{1}{2\varepsilon^{2}}\ln\frac{1-\gamma }{2}, \end{array}$$

where *ε* is the maximal level of error that can be accepted by *u*_*k*_, and *γ* is the confidence measure. Note that when *ω*_2_ = 1, the agent consider only direct trust part. In order to more accurately model the advisor's trustworthiness, direct connections and contagious influence will always be used together. That is, in (4) and (5), if *ω*_2_ + *ω*_3_ > 1, then we change the weights to be *ω*_2_ = *ω*_2_/(*ω*_2_ + *ω*_3_) and *ω*_3_ = 1 − *ω*_2_. *ω*_1_ = 1 − *ω*_2_ − *ω*_3_.

### 2.3. The Improved Probabilistic Factor Model Based on Trust Diffusion Theory

**Figure 3:**
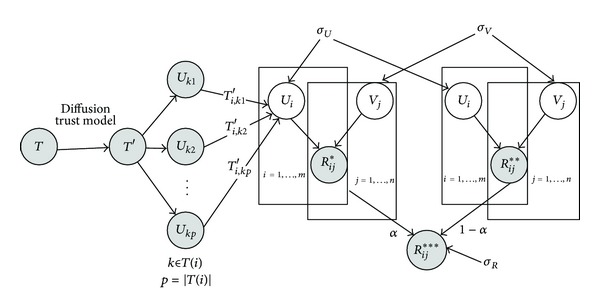
The improved probabilistic factor graph based on trust diffusion model.

In this section, we present an improved model in Figure 3 (DiffTrust + PMF) based on the social trust ensemble (RSTE) [8]. RSTE is based on the following three intuitions. (1) Users have their own characteristics, and they have different tastes on different items. (2) Users can be easily influenced by the friends they trust and prefer their friends' recommendations. (3) One user's final decision is the balance between his/her own taste and his/her trusted friends' favors. According to these intuitions, the rating *R*_*ij*_ in the user-item matrix is interpreted as the representation mixed by both the user *u*_*i*_'s taste and his/her trusted friends tastes on the item *v*_*j*_. RSTE works to some extent and further has other inherent weaknesses. For example, RSTE only deals with the explicit trust in social trust network. However, trust network from preexisting social links between users is rather sparse, which leads to the the fact that direct trust relationships can only play a small role in the process of the recommendation. Different from RSTE, our improved method integrates diffusion trust model into probabilistic factor graph, which will match more trust users for the current user through DiffTrust model, fully dig up more new trust relationships between users, and is used for recommendation service. In other words, our method not only combines the users' tastes with their trusted friends' favors for recommendation, but also studies systematically the implicit relationship of the trust network based on diffusion theory. DiffTrust + PMF can help to alleviate the trust network sparsity problem and will potentially increase the recommendation accuracy. As shown in Figure 3, the left part is trust diffusion model (DiffTrust) and the right part is the social network matrix factorization model. We first employ DiffTrust to explore the processes in which the trust is cultivated. The DiffTrust can be used to infer the implicit relationship of the trust network. The original matrix *T* is enriched into the dense matrix *T*′ (the detailed process is introduced in Section 2.2). *T*(*i*) is the set of users who are trusted by user *u*_*i*_ in the trust network, and |*T*(*i*)| is the number of trusted friends of user *u*_*i*_ in the set *T*(*i*). Next, we model the problem of social recommendation using matrix factorization model, which makes his/her own taste and his/her trusted friends' favors together. That is, one user's final decision is the balance between his/her own taste and his/her trusted friends' favors. Based on Figure 3, the conditional distribution over the observed ratings and trust relationships is given by

(8) p(U,V ∣ R∗∗∗,T′,σR2,σU2,σV2)=∏i=1m∏j=1n[N(Rij ∣ g(αUiTVj+(1−α)∑k∈T(i)Tik′UkTVj),          σR2)]IijR∗∗∗  ×∏i=1mN(Ui ∣ 0,σU2I)×∏j=1nN(Vj ∣ 0,σV2I),

where *N*(*x* | *μ*, *σ* ^2^) is the probability density function of the Gaussian distribution with mean *μ* and variance *σ* ^2^, and *I*_*ij*_^*R*∗∗∗^ is the indicator function that is equal to 1 if user *u*_*i*_ rated item *v*_*j*_ and equal to 0 otherwise. The function *g*(*x*) is the logistic function *g*(*x*) = 1/(1 + exp⁡(−*x*)), which makes it possible to bound the range *U*_*i*_^*T*^ *V*_*j*_ from within the range [0,1]. The parameter *α* is used to smooth the users' favors and the trusted friends' favors, which controls how much users trust themselves or their trusted friends.

The log of the posterior distribution for the recommendations is given by

(9) ln⁡p(U,V ∣ R∗∗∗,T′,σR2,σU2,σV2)=−12σR2 ×∑i=1m∑j=1nIijR∗∗∗(Rij∗∗∗−g(αUiTVj+(1−α)∑k∈T(i)Tik′UkTVj))2 −12σU2∑i=1mUiTUi−12σV2∑j=1nVjTVj −12(∑i=1m∑j=1nIijR)ln⁡σ2−12(mlln⁡σU2+nlln⁡σV2)+C,

where *C* is a constant that does not depend on the parameters. Maximizing the log-posterior over two latent features with hyper-parameters (i.e., the observation noise variance and prior variances) kept fixed is equivalent to minimizing the following sum-of-squared-errors objective functions with quadratic regularization terms:

(10) L(R,T′,U,V) =12∑i=1m∑j=1nIijR∗∗∗(Rij∗∗∗−g×(αUiTVj+(1−α)∑k∈T(i)Tik′UkTVj))2  +λU2||U||F2+λV2||V||F2,

where *T*(*i*) is the set of users who are trusted by user *u*_*i*_ in the trust network, *λ*_*U*_ = *σ* ^2^/*σ*_*U*_^2^, and *λ*_*V*_ = *σ* ^2^/*σ*_*V*_^2^. In order to reduce the model complexity, in all of the experiments we conduct in Section 4, we set *λ*_*U*_ = *λ*_*V*_. ||·||_*F*_^2^ denotes the Frobenius norm, ||*U*||_*F*_^2^ = ∑_*i*=1_^*m*^ *U*_*i*_^*T*^ *U*_*i*_ and ||*V*||_*F*_^2^ = ∑_*j*=1_^*n*^ *V*_*j*_^*T*^ *V*_*j*_.

A local minimum of the objective function given by (8) can be found by performing gradient descent in *U*_*i*_, *V*_*j*_,

(11) ∂L∂Ui=α∑j=1nIijR∗∗∗g′(αUiTVj+(1−α)∑k∈T(i)Tik′UkTVj)Vj×(g(αUiTVj+(1−α)∑k∈T(i)Tik′UkTVj)−Rij∗∗∗)(1−α)∑q∈B(i) ∑j=1nIqjR∗∗∗g′  ×(αUqTVj+(1−α)∑k∈T(i)Tik′UkTVj)  ×(g(αUqTVj+(1−α)∑k∈T(i)Tik′UkTVj)−Rqj∗∗∗)  ×Tqj′Vj+λUUi,∂L∂Vj=∑i=1mIijR∗∗∗g′(αUiTVj+(1−α)∑k∈T(i)Tik′UkTVj)×(g(αUiTVj+(1−α)∑k∈T(i)Tik′UkTVj)−Rij∗∗∗)×(αUi+(1−α)∑k∈T(i)Tik′UkT)+λVVj,

where *g*′(*x*) is the derivative of logistic function *g*′(*x*) = exp⁡(*x*)/(1 + exp⁡(*x*)^2^), and *B*(*i*) is the set of the users who trust user *u*_*i*_.

## 3. Experimental Evaluation

In this section, we firstly introduce datasets and evaluation metric. Then we compare performance of our novel method.

### 3.1. Dataset Description

We evaluate our method on Epinions [22], Flixster [23], and Douban [24] datasets.

*Epinions* dataset [22] was given directly by Epinions staff to Paolo Massa. Note that it is not a typical collaborative filtering dataset, since the ratings are about the articles and not about items: the ratings represent how much a certain user rates a certain textual article written by another user, that is, a review. We randomly select 2,100 users and 3,648 articles. These users issued 6408 article ratings and 5,392 statements (4,575 trusts and 817 distrusts).

*Flixster* [23] is a social movie site allowing users to share movie ratings, discover new movies and meet others with similar movie taste. This contains the friendship network crawled in December 2010 by Javier Parra (Javier.Parra@asu.edu). We randomly select 1,000 users and 2,913 items from the Flixster dataset. These users issued 8,127 ratings and 3,714 relation statements.

*Douban* dataset, crawled by Ma et al. [24], contains 16,830,839 ratings of 129,490 users on 58,541 movies and 1,692,952 friend links between these users. The Douban dataset used in our experiments consists of 3,160 users who have rated at least one of a total of 6,529 different items. The total number of ratings is 12,963 and the total number of friend links is 7,152.

The statistical information of these three datasets is summarized in Table 1.

### 3.2. Evaluation Metrics

We measure the prediction quality of our proposed method in terms of the root mean square error (RMSE), Recall, and *F* Measure.

The metrics RMAE is defined as

(12) $$\begin{array}{c} \text{RMSE}=\sqrt{\frac{1}{N}\underset{i,j}{\sum }{\left(r_{i,j}-{\hat{r}}_{i,j}\right)}^{2}}, \end{array}$$

where *r*_*i*,*j*_ and ${\hat{r}}_{i,j}$ denote the actual and recommended rating user *i* gave to item *j*, respectively. *N* denotes the number of tested ratings. The smaller the value of RMSE, the more precise the recommendation.

Recall is the percentage of 〈*user*, *item*〉 pairs in the test set for which we can compute a recommendation.

Consider

(13) $$\begin{array}{c} \text{Recall}=\frac{tp}{tp+fn}, \end{array}$$

where *tp* is the number of actual 〈*user*, *item*〉 pairs predicted by recommendation method and *fn* is the number of actual 〈*user*, *item*〉 pairs not predicted by recommendation method.

We compute the *F* Measure by combining RMSE and Recall into a single evaluation as follows:

(14) $$\begin{array}{c} F\;\;\text{Measure}=2\times \frac{\text{Precision}\times \text{Recall}}{\text{Precision}+\text{Recall}}, \end{array}$$

where the precision is defined as follows:

(15) $$\begin{array}{c} \text{Precision}=1-\frac{\text{RMSE}}{4}. \end{array}$$

In this equation, 4 is the maximum possible error since the values of ratings are in the range [1,5].

### 3.3. Comparison of the Primary DiffTrust and Novel DiffTrust

**Figure 4:**
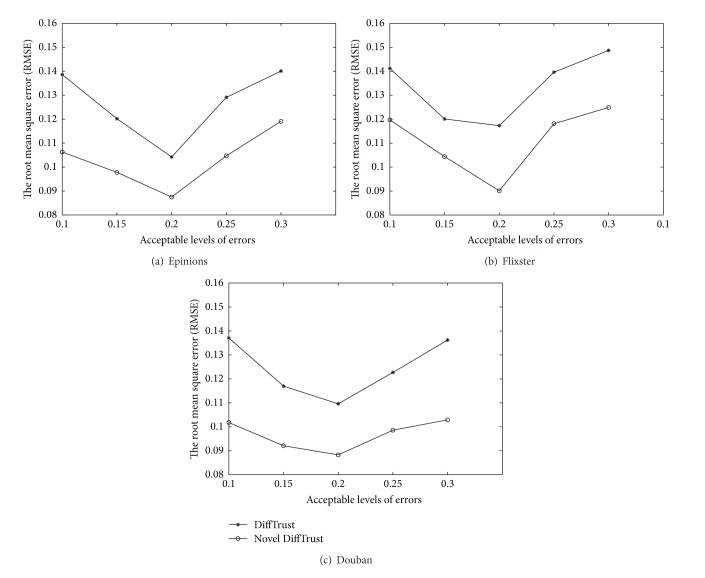
Impacts of *ε* on RMSE.

To validate and gain insights about the usefulness of our novel DiffTrust introduced in Section 2.2. For all three datasets, we use the metrics, the root mean square error (RMSE), between predicted trust value for each user pair and the actual trust relationship, to measure the prediction quality of our method in comparison with the existing DiffTrust [15]. In all the experiments conducted in the following sections, we adopt the method in [21] to set the parameter *γ* = 0.8. It means that the confidence value should be no less than 0.8 in order for the users to be confident about their own evaluation values of advisors. In addition, we set the time decay factor *λ* = 0.8 (in (1) and (5)) according to the paper [25]; when *λ* = 0.8, the model reaches its best performance.  Table 2 lists different acceptable levels of errors, correspondent weights from direct connections, and different results of RMSE.  Figure 4 shows the corresponding charts.

From Figures 4(a), 4(b), and 4(c), it is clear that our novel DiffuTrust performs quite well compared with the primary DiffTrust. This is probably because our improved method allows exponential decline in the additive model, which better accommodates the introduction of temporal heterogeneity. In addition, we observe that the value of *ε* impacts the results of RMSE significantly. No matter our novel DiffuTrust or primary DiffTrust, as *ε* increases, RMSE decreases (prediction accuracy increases) at first, but when *ε* surpasses a certain threshold, RMSE increases (prediction accuracy decreases) with further increase of the value of *ε*. This phenomenon confirms the intuition that purely using the effect from intrinsic tendencies or purely using the effect from direct connections or purely using contagious influence to model the advisor's trustworthiness cannot generate better performance than fusing these three favors together. It should be noted that both the two methods achieve the best performance when *ε* = 0.2, while smaller values like *ε* = 0.15 or larger values like *ε* = 0.25 can potentially degrade the model performance. These experiments clearly demonstrate different acceptable levels of errors (for *u*_*k*_) can impact the advisor's trustworthiness.

### 3.4. Results and Analysis

In this section, in order to show the performance improvement of our DiffTrust + PMF method, we compare our method with the following methods.

 
*PMF* [12]—probabilistic matrix factorization, it is a probabilistic linear model with Gaussian observation noise and only uses user-item matrix for the recommendations.
 
*BPMF* [13]—Bayesian probabilistic matrix factorization, it provides a predictive distribution instead of just a single number, allowing the confidence in the prediction to be quantified and taken into account when making recommendations using the model.
 
*RSTE* [8]—recommendation with social trust ensemble (RSTE), it is proposed by Ma et al. and employs opinions of the trusted friends in the social trust network to make recommendations for the users.

**Figure 5:**
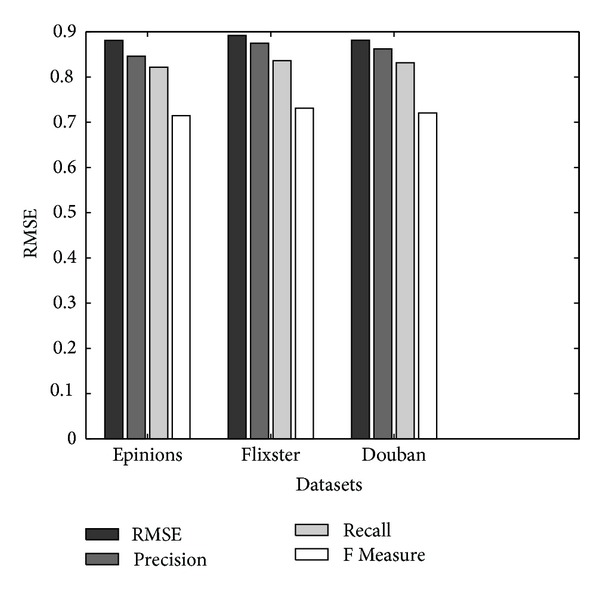
Comparison of RMSE for different methods.

**Figure 6:**
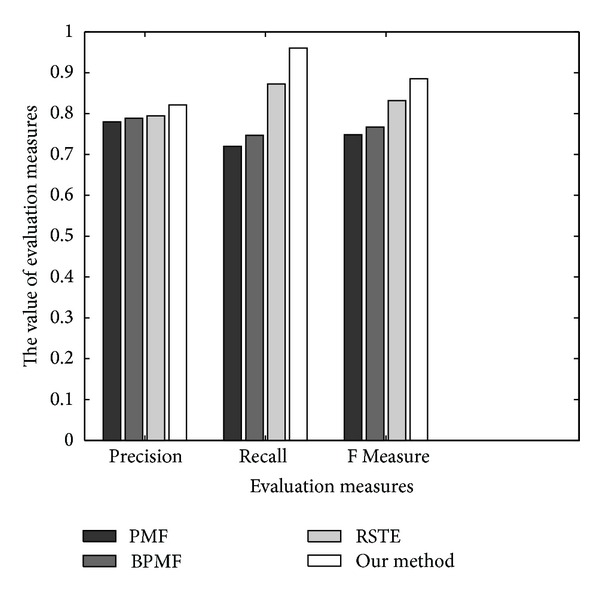
Comparison of evaluation measures for different methods on Epinions Dataset.

The parameter setting of our method is *α* = 0.4, and in all the experiments conducted in the following sections, we set all of the parameters *λ*_*U*_ = *λ*_*V*_ = 0.001.  Table 3 shows the RMSE, Coverage, and *F* Measure for all comparisons. Figures 5 and 6 show the charts comparing the results of different methods according to each of the three evaluation measures separately.

As shown in Figure 6, our method and RSTE have lower error than the other two methods. This is probably because our method and RSTE take into account the users' tastes with their trusted friends' favors for recommendation. In addition, the RMSE of our method's RMST (0.7146) is lower than that of RSTE (0.8219), which means that considering the possible diffusions of trusts between various users can reduce the error. Figure 6 shows the *F* Measure together with precision and coverage for all methods on Epinions dataset. Our method outperforms all other methods in terms of *F* Measure. It should be noted that our method not only clearly has a lower error, but also has a better coverage than other all methods on the three datasets.

In summary, our method clearly outperforms matrix factorization for recommendation (both PMF and BPMF) in terms of coverage because of exploiting the trust-network. Moreover, our method substantially improves the precision of existing trust-based method (RSTE). This improvement is achieved by considering trust diffusion processes. This consideration will help to alleviate the data sparsity problem and will potentially increase the prediction accuracy.

## 4. Related Work

Here we categorize the related works into two areas: trust diffusion and recommender systems.

Our recommendation method is based on trust diffusion. Trust diffusion, which is also known as trust propagation, is about predicting the trust worthiness of nonadjacent agents by combining trust values through distinct indirect paths. Trust propagation is widely studied in [26–28]. Teacy et al. [26] develop TRAVOS, which models an agent's trust in an interaction partner. Specifically, trust is calculated using probability theory taking account of past interactions between agents. Hang et al. [27] model trust as a binary event. They define three operators for concatenating trust along a path, aggregating trust from distinct paths from the same witness, and selecting the most trustworthy path among all witnesses, respectively. Teacy and Hang only consider the user's own experience with the advisor, which may lead to inaccurate trust evaluation when the user has only limited experience with the advisor or the advisor dynamically changes her behavior. To address this problem, Fang et al. [28] propose a trust diffusion model; trustworthiness perceived by a specific user is measured under a specific context. Their method can flexibly model adjusts the weight of the user's own experience and other users' evaluation on the advisor. In order to better accommodate the introduction of temporal heterogeneity, we improve the method in paper [28]. Our improved DiffTrust allows exponential decline in the additive model, the experimental results show that our novel DiffTrust performs quite well compared with the primary DiffTrust. Some other trust propagation methods [18, 29, 30] have also been proposed.

Now we discuss some related work of recommender systems. Recommender system is an indispensable technique in the field of information filtering. Trust-based recommendation assumes the additional knowledge of a trust network among users and can better deal with cold start users. Most related to our method, Ma et al. [8] utilized a novel probabilistic factor analysis framework to fuse the users' tastes and their trusted friends' favors together by an ensemble parameter: their method achieves an improvement over the very large datasets since it scales linearly with the number of observations. Different from their method, we mainly consider the possible diffusions of trusts between various users by combining DiffTrust with PMF for recommendation, which can help to alleviate the trust network sparsity problem and will potentially increase the prediction accuracy. Other similar works are also employing trust relationship among users to solve recommendation problems by [11, 31–33]. For example, [11] proposed a novel implicit trust aware recommendation model (iTARS) based on the small-worldness of the implicit trust network, in which the implicit trust is generated from the user similarities. Wang et al. [31] develop a method to generate trust between users based on their common tastes. By first grouping items into different clusters, the authors use a frequency measure of the number of ratings each user asserts to different groups of items, and thus build up a personalized taste set for user and infer trust based on the common taste sets between users. Shambour and Lu [32] propose a fusion-based recommendation approach that fuses the trust and semantic information of users and items within the CF framework to achieve yet more effective results in terms of recommendation accuracy and coverage, especially when dealing with “data sparsity” and “cold start” problems. To sum up, most of these trust-based recommendation algorithms have exposed one major limitation: they do not consider trust network dynamic and evolutionary (varying over time or interaction context). This problem has further been addressed in our work.

Other algorithms based matrix factorization have been proposed for recommendation, which assume that the preference of a user can be represented by a small number of unobserved features, such as probabilistic matrix factorization (PMF) [12], variational Bayesian matrix factorization (VBMF) [34], Bayesian probabilistic matrix factorization (BPMF) [13], and general probabilistic matrix factorization (GPMF) [35]. However, these methods suffer from the “data sparsity” problem and the “cold start” problem. These problems have further been addressed in our work by fusing social relations among users with rating data, which can help to improve the performance of recommender systems.

## 5. Conclusion

In our study of recommendation method, we found two prevalent problems which must be resolved in order to make a more useful recommendation. The first problem regards dealing with the possible diffusions of trust between various users and the second is the data sparsity problem. As discussed earlier, the challenge arises in finding the ideal tradeoff between the accuracy and efficiency, which is the focus in this paper.

In this study, we have presented the DiffTrust + PMF method and achieved good results in recommendation. We have (1) significantly studied the implicit relationship of the trust network based on diffusion theory and alleviated the data sparsity problem, and (2) made use of the users' tastes and their trusted friends' favors for recommendation by integrating diffusion trust model into probabilistic factor graph. Experimental results based on real datasets showed that our method of combining DiffTrust with the PMF can achieve high performance.

In this paper, we only use the trust information, while distrust statements are also provided in many online social networks. The future work intends to apply distrust information to recommendation systems. An important problem worthy of future research is how the distrust relations affect the user preference.

## Figures and Tables

**Table 1:** Statistical information about the three real datasets.

Datasets	Epinions	Flixster	Douban
Statistics	2,100 users	1,100 users	3.160 users
	3,648 articles	2,913 movies	6,529 movies
	6,408 article ratings	8,127 movie ratings	12,963 movie ratings
	5,392 relations statements	3,714 relation statements	7,152 relation statements

Data types	users → articles	users → movies	users → movies
	users → users	users → users	users → users

Rating scale	1, 2, 3, 4, 5	0.5,1, 1.5,…, 5	1, 2, 3, 4, 5

Trust between users	1	1	1

**Table 2:** Shows the impacts of ε on RMSE.

Datasets	ε	0.1	0.15	0.2	0.25	0.3
	*N*_min⁡_	115	51	29	19	13
Epinions	RMSE (DiffTrust)	0.1386	0.1202	** 0.1042 **	0.1291	0.1401
	RMSE (Novel DiffTrust)	0.1063	0.0978	** 0.0875 **	0.1047	0.1191

Flixster	RMSE (DiffTrust)	0.1412	0.1201	** 0.1173 **	0.1396	0.1487
	RMSE (Novel DiffTrust)	0.1197	0.1044	** 0.0901 **	0.1181	0.1249

Douban	RMSE (DiffTrust)	0.1371	0.1170	** 0.1096 **	0.1227	0.1362
	RMSE (Novel DiffTrust)	0.1018	0.0921	** 0.0883 **	0.0986	0.1029

**Table 3:** Shows all comparisons on three datasets.

Datasets	Metrics	PMF	BPMF	RSTE	Our method
Epinions	RMSE	0.8812	0.8461	0.8219	**0.7146**
	Precision	0.7797	0.7885	0.7945	**0.8213**
	Recall	0.7196	0.7468	0.8725	**0.9603**
	*F*Measure	0.7484	0.7671	0.8317	**0.8854**

Flixster	RMSE	0.8919	0.8749	0.8362	**0.7313**
	Precision	0.7770	0.7813	0.7910	**0.8172**
	Recall	0.6817	0.7364	0.8449	**0.9415**
	*F* Measure	0.7262	0.7582	0.8170	**0.8749**

Douban	RMSE	0.8815	0.8625	0.8317	**0.7207**
	Precision	0.7796	0.7844	0.7921	**0.8200**
	Recall	0.7024	0.7409	0.8616	**0.9571**
	*F* Measure	0.7390	0.7620	0.8254	**0.8832**
